# Rapid Implementation of Pulse Oximetry Newborn Screening to Detect Critical Congenital Heart Defects — New Jersey, 2011

**Published:** 2013-04-19

**Authors:** Lorraine F. Garg, Mary M. Knapp, Leslie M. Beres, Kim Van Naarden Braun, Cynthia F. Hinton, Cynthia H. Cassell, Richard S. Olney, Cora Peterson, Jill Glidewell

**Affiliations:** New Jersey Dept of Health; Div of Birth Defects and Developmental Disabilities, National Center on Birth Defects and Developmental Disabilities; EIS officer, CDC

In August 2011, New Jersey implemented a statewide newborn screening protocol for critical congenital heart defects (CCHD) using pulse oximetry. In January 2012, CDC responded to a request from the New Jersey Department of Health (NJDOH) to assist with an assessment of the implementation. Out of the 52 birthing facilities in New Jersey, a sample of 11 was selected. Staff interviews were conducted to assess screening and data collection processes, data flow and tracking procedures, electronic medical record (EMR) capabilities, and capacity to report data to NJDOH. Feedback also was obtained about the questionnaire being used to follow-up on positive screening results. All 11 facilities were screening for CCHD. Among the 11 facilities, three were electronically entering and maintaining data into an EMR, five were manually entering and maintaining data into paper charts and logs, and three were both electronically and manually entering and maintaining data. Facilities reported that implementation of newly mandated CCHD screening posed a low burden to hospital staff members. NJDOH receives aggregate pulse oximetry screening data from all New Jersey birthing facilities. During the first 3 months of screening, preliminary data indicated that 98.2% of 25,214 newborns were screened. Hospitals reported data on 12 newborns with positive screening results; two newborns were newly diagnosed with CCHD as a result of pulse oximetry screening. Because of state-specific factors, such as out-of-state referral patterns, these findings might underestimate the anticipated number of positive screens in states with varying referral patterns and use of prenatal diagnosis. Rapid implementation of universal CCHD screening posed a relatively low burden to hospitals in New Jersey.

The system assessment began in January 2012 (5 months after hospitals commenced routine screening). The objectives were to assess EMR capabilities, assess the capacity to report screening data to NJDOH, and evaluate the data flow and tracking at a sample of birthing facilities. As part of this investigation, 11 of 52 New Jersey birthing hospitals were visited. Four of these birthing facilities were included because they had identified newborns with positive screening results during the first 3 months of implementation. The other seven hospitals were selected as a random sample of all other birthing facilities in the state, stratified by geographic location, hospital birth census, and hospital level of care.

NJDOH’s mechanism for pulse oximetry surveillance includes collection of aggregate data reports from each licensed birthing facility and reports of positive screening results to the confidential New Jersey Birth Defects Registry (NJBDR). The aggregate data reports submitted to NJDOH contain the number of live births, number of newborns screened, an explanation of any discrepancies between those numbers, and the number of positive screens. During the investigation, NJDOH staff members shared results of these preliminary aggregate screening data from the first 3 months of system operation (August 31–November 30, 2011).

A structured questionnaire with open-ended questions was developed by the investigation team and distributed to hospitals before the field investigation. Face-to-face interviews were conducted by CDC and NJDOH personnel to assess pulse oximetry screening procedures, data collection and maintenance procedures, reporting practices, and burden (i.e., increased workload or additional duties) of screening and reporting by hospital staff. Staff members were asked to rate the level of burden on a scale ranging from 1 = no burden to 10 = very burdensome. Key personnel, such as well baby nursery and neonatal intensive care unit managers and staff nurses, clinical educators, and representatives from the hospital’s biomedical services department, participated in each interview. Medical charts were reviewed with hospital staff members at the four facilities that previously had reported positive screens and the process of reporting data to NJDOH was discussed. Feedback on the questionnaire being used for follow-up of positive screens was obtained from facility staff members. Members of the investigation team observed pulse oximetry screening and documentation practices in each hospital’s well baby nursery and neonatal intensive care unit.

All 11 hospitals had incorporated screening for CCHD into routine nursing care in their well baby nurseries and neonatal intensive care units. Hospital nurses reported that the addition of the newly mandated screening processes posed minimal burden (average score 2.1). Nurses indicated that pulse oximetry was a familiar skill and screening all newborns was easily incorporated into their routine tasks. Three of the 11 hospitals were electronically entering and maintaining data in an EMR, five of the 11 were manually entering and maintaining data into paper charts or logs, and three of the 11 were both electronically and manually entering and maintaining data. All facilities had mechanisms for collecting and reporting aggregate screening data to NJDOH and positive screening results to NJBDR. All facilities reported that aggregate screening data would be submitted to NJDOH as requested. Hospitals reported the process of submitting aggregate screening data to NJDOH posed a moderate burden (average score 4.2) to staff members. Hospitals requested a form with detailed instructions to report discrepancies between the number of live-births and number of newborns screened for future aggregate screening data requests. All facilities reported that individual-level screening and clinical data would be reported to NJBDR for positive screening results. The NJBDR follow-up questionnaire was modified based on feedback from nurses.

In the first 3 months following implementation of the mandate to screen all newborns for CCHD using pulse oximetry, preliminary data indicated that 98.2% of 25,214 newborns born in licensed birthing facilities were screened, with 12 positive screens. Two positive screens were confirmed CCHD cases initially detected by pulse oximetry screening (no prior diagnosis), which otherwise might have resulted in death or disability.

## Editorial Note

Universal newborn screening is the process by which newborns are screened shortly after birth for conditions that can cause severe illness, disability, or death. Through early identification and treatment, newborn screening provides an opportunity to reduce morbidity and mortality (1). The Recommended Uniform Newborn Screening Panel is a list of conditions for which all newborns should be screened, as recommended by the U.S. Secretary of Health and Human Services.* In September 2011, the Secretary approved the addition of screening for critical congenital heart disease using pulse oximetry to the panel.† Congenital heart disease occurs in nearly 1% of live births; approximately one quarter of cases will be CCHD, defined as those defects requiring cardiac surgery or catheterization before age 1 year (2,3).

Pulse oximetry is a noninvasive technology that can be used to detect hypoxemia, a clinical sign of CCHD (2). In the absence of early detection, newborns with CCHD are at risk for death in the first few days or weeks of life. Current approaches for detection of CCHD include prenatal ultrasound or physical examination findings (e.g., cyanosis or tachypnea), although not all infants with CCHD are detected with prenatal ultrasound screening or by physical examination findings (2,3). Predictive values and sensitivity of pulse oximetry screening varies based on the screening protocol used (e.g., timing of screening after birth or number of extremities measured) (4). On June 2, 2011, New Jersey passed legislation requiring that all licensed birthing facilities screen newborns for CCHD at age ≥24 hours using pulse oximetry.§ This legislation became effective 90 days later, on August 31, 2011, making New Jersey the first state to implement mandatory statewide CCHD screening. This report represents the first systematic gathering of process data on legislatively mandated newborn screening for CCHD in the United States. Most states have not yet mandated universal newborn screening for CCHD; however, similar legislation was introduced or enacted in at least 18 other states during their 2011–2012 legislative sessions (American Academy of Pediatrics, Division of State Government Affairs, unpublished data, 2012).

What is already known on this topic?

Congenital heart disease occurs in approximately 1% of live births, and approximately one quarter of cases will be critical congenital heart defects (CCHD). Newborn screening using pulse oximetry can be used to detect hypoxemia, a clinical sign of CCHD. In August 2011, New Jersey implemented a statewide newborn screening protocol for critical congenital heart defects using pulse oximetry.

What is added by this report?

Five months after the CCHD screening program was implemented in New Jersey, all hospitals in a sample were screening and reporting data to the state health department. Hospitals reported that implementation of the newly mandated pulse oximetry screening posed minimal burden (i.e., increased workload or additional duties) to their nursing staff members. During the first 3 months of screening, two newborns were identified through screening as having previously unsuspected CCHD.

What are the implications for public health practice?

Rapid implementation of universal CCHD screening posed a relatively low burden to hospitals in New Jersey. Data collection and reporting are essential to evaluate the effect of this public health program.

New Jersey birthing facilities were willing and capable of screening, collecting data, and reporting data to NJDOH. Reporting of aggregate birthing facility screening data to NJDOH is a short-term measure until pulse oximetry screening results can be incorporated into an electronic birth reporting system. For this short-term measure, CDC recommended that NJDOH provide a prescriptive form for future aggregate data requests. All facilities in the sample had incorporated screening and documentation of results. Some facilities had more sophisticated methods of data maintenance and tracking to ensure that all newborns are screened.

In the first 3 months of screening, two newborns were detected with CCHD using pulse oximetry that otherwise might have resulted in death or disability. Pulse oximetry screening also might identify other medical conditions, such as pulmonary conditions or sepsis, potentially improving newborn care and subsequent outcomes. New Jersey–specific characteristics, such as mothers at high risk choosing to deliver in birthing facilities in surrounding states, might have influenced the number of positive screens that were reported. These and other factors should be considered before using New Jersey data to estimate resource needs.

## Footnotes

* Available at http://www.hrsa.gov/advisorycommittees/mchbadvisory/heritabledisorders/recommendedpanel/index.html.

† Additional information available at http://www.hrsa.gov/advisorycommittees/mchbadvisory/heritabledisorders/recommendations/correspondence/cyanoticheartsecre09212011.pdf.

§ Birthing facilities required to perform pulse oximetry screening; rules, regulations, Pub. L. 2011 Chapter 74 (State of New Jersey, 2011). Available at http://www.njleg.state.nj.us/2010/bills/al11/74_.htm.
